# Isolation of nematophagous fungi from soil samples collected from three different agro-ecologies of Ethiopia

**DOI:** 10.1186/s12866-022-02572-4

**Published:** 2022-06-17

**Authors:** Maradona Berhanu, Hika Waktole, Gezahegne Mamo, Getachew Terefe

**Affiliations:** 1 Alage Agricultural Technical and Vocational Educational Training College, Addis Ababa, Ethiopia; 2 Department of Microbiology, Immunology and Veterinary Public Health, College of Veterinary Medicine and Agriculture, Addis Ababa University, Bishoftu, Ethiopia; 3 Department of Pathology and Parasitology, College of Veterinary Medicine and Agriculture, Addis Ababa Bishoftu, Ethiopia

**Keywords:** Nematophagous fungi, Nematode, Agroecology, Soil

## Abstract

**Background:**

Several species of nematophagous fungi exist in nature that can capture and kill nematodes as natural predators of soil-dwelling worms. These are important in agriculture and animal husbandry as biological control agents. The diversity of nematophagous fungi found from soil had not been studied in Ethiopia.

**Objective:**

This study aimed to isolate Nematophagous Fungi from Soil Samples Collected From three Different Agro-Ecologies of Ethiopia.

**Methods:**

Cross-sectional study was conducted and samples were collected from three different agro-climatic zones of Ethiopia; Debre-Berhan (highland), Bishoftu (mid-altitude), and Awash (lowland). Twenty-seven soil samples were randomly taken from each of the three different agro-ecological climates (9 from each agro-ecological climatic zone). For each study site, samples were collected from the soil of decomposed animal feces/dung, agricultural/farmlands, and forest lands in triplicates.

**Results:**

The present study disclosed that nematophagous fungi were widespread from the study area. A total of 33 species of nematophagous fungi belonging to four genera, *Arthrobotryes, Paecilomyces, Monacrosporium*, and *Harposporium* were identified. *Arthrobotrys* were the most commonly isolated genera followed by *Paecilomyces*. The six identified species were *Arthrobotrys oligospora, Paecilomyces lilacinus, Arthrobotryes dactyloides, Monacosporum eudermatum, Harposporium helicoides,* and *Monacosporum cionopagum.*

**Conclusion:**

This study indicated that *Arthrobothryes oligospora* was the most common species in Bishoftu and Awash whereas. In Debre-Berhan, *Paecilomyces lilacinus* was the *most* prevalent species. *Monacosporum cionapagum* was not isolated from dung soil and agricultural soil whereas *Harposporium helicoides and Arthrobothryes dactyloides were* not found from dung and forest soil respectively.

**Supplementary Information:**

The online version contains supplementary material available at 10.1186/s12866-022-02572-4.

## Background

Gastrointestinal nematodes in grazing animals produce significant productivity losses and are a worldwide animal welfare issue. Repeated use of anthelmintics to control helminth infection frequently led to the development of drug resistance [1]. Resistance to all classes of broad-spectrum anthelmintics has already been reported [2]. As time has passed problems of multi-resistance to more than one class have occurred and the multi-resistant nematode has become a major threat to the whole small ruminant industry [3].

Due to the failure of anthelmintic drenches, many scholars have been underway for the past 25 years to have alternatives to chemical control [4]. The demand for the development of alternatives such as biological control agents is expanding for both animal and plant helminth pathogens [5]. The use of biological control methods such as nematode-trapping fungi, diets high in condensed tannins and other plant materials, as well as other nutritional approaches have all been examined as possible approaches to reduce the impact of nematode parasites in domestic livestock [4].

Biological control is a method in which biological agents can be used to reduce the population of parasites either on pasture or in the host and by so doing minimize the frequency of anthelmintic usage [6]. One example of biological control against gastrointestinal nematodes is the use of some species of soil-derived fungi (Nematophagous fungi). These fungi have the potential to reduce nematode larval populations on pasture by using these either as their main source of nutrients or as a supplement to a saprophytic existence [7]. Natural feeders of gastrointestinal nematodes, nematophagous fungus, have received a lot of attention because of their potential importance as biological control agents for nematodes that parasitize plants, animals, and humans [8–10]. They are micro-fungi that can capture, kill, and digest nematodes [11] and hence are natural predators of soil-dwelling nematodes [12].

Based on their mechanism of action or killing process Nematophagus fungus is grouped into four classes (endoparasitic fungi, nematode-trapping fungi, egg parasitizing fungi, and toxin-producing fungi) [13–15]. Nematophagous fungi have the potential to reduce pasture contamination by reducing the numbers of larvae in and around dung due to their ability to kill nematodes in the niches occupied by juvenile (larval) stages of parasites [16]. They are found in terrestrial and aquatic environments. However, they are mostly concentrated in the upper part of the soil, in pastures, leaf litter, mangroves, and certain shallow water bodies [17, 18]. Therefore, it can be relatively easy to isolate nematophagous fungi, particularly from soils and organic matter.

Despite the aforementioned advantages of Nematophagus fungi and as far as available literature is concerned, no study has ever attempted in Ethiopia to isolate and characterize nematophagous fungi, which can be used as potential biological agents against helminth parasites of livestock. Therefore, this study aimed to isolate Nematophagous Fungi from Soil Samples Collected From three Different Agro-Ecologies of Ethiopia.

## Methods

### Study area and period

**Fig. 1:**
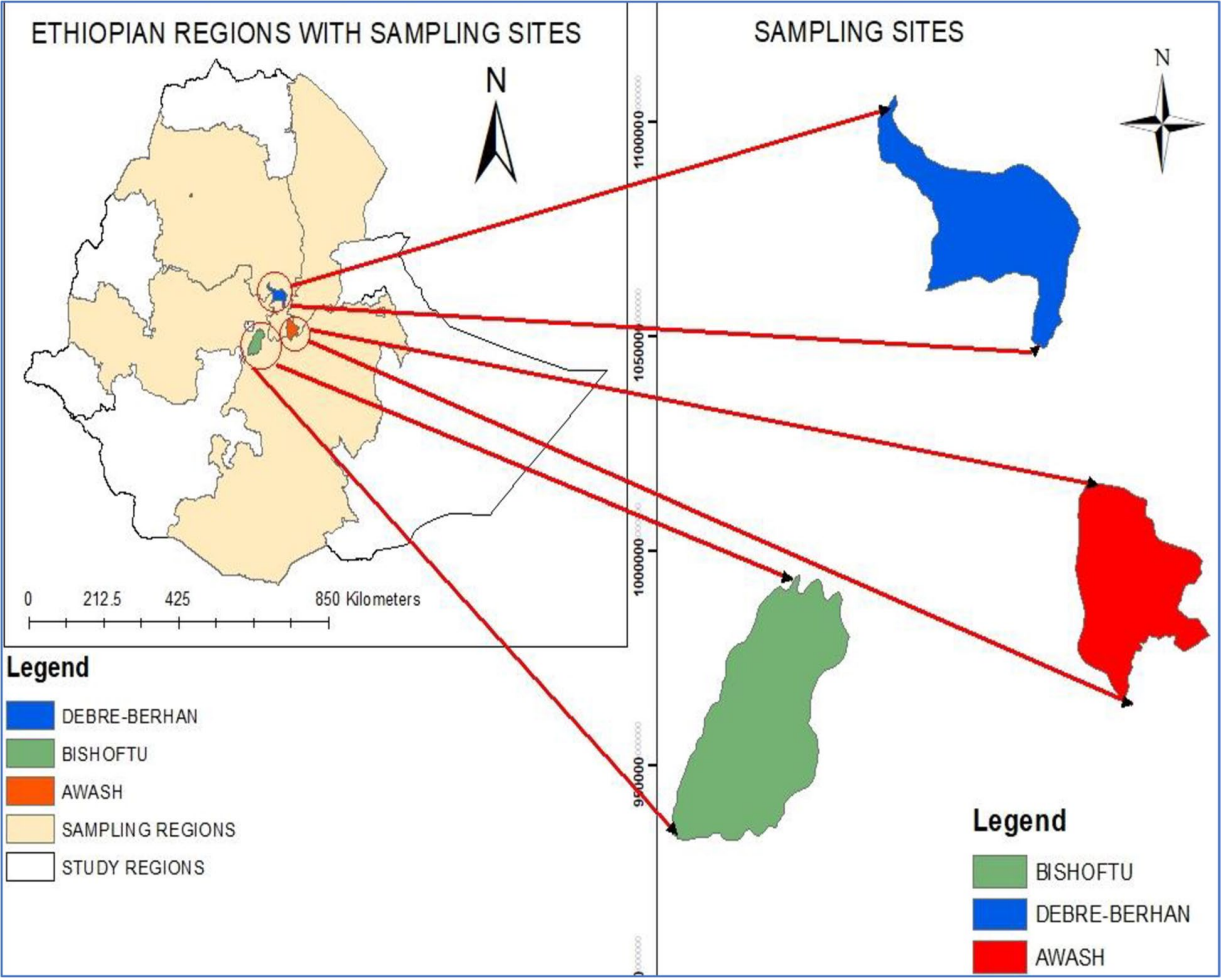
Map of a study area

Samples for this research work were collected from three different agro-climatic zones of Ethiopia; Debre-Berhan (highland), Bishoftu (mid-altitude), and Awash (lowland) (Fig. 1). Debre-Berhan is the city of north Shewa zone of the Amhara region. It is about 120 km northeast of Addis Ababa with an altitude of 2,780 m above sea level. This is a mountainous area dissected by rivers and streams. The average annual rainfall in the region is 920 mm, while the average monthly minimum and maximum ranges of air temperature vary between 2.4 °C and 8.5 °C and 18 °C to 23.3 °C respectively. The average soil temperature at 5 cm depth is 13.6 °C. The second study area, Bishoftu, is found around 44 km southeast of Addis Ababa and it has an elevation of about 1,878 m above sea level (mid-altitude). The area experiences a bimodal rainfall pattern with a short rainy season from February to April and the long rainy season from the middle of June to the end of September. The remaining months are dry periods. The area gets an annual average rainfall of 892 mm and the average annual temperature is 18.7 °C. The third study site, Awash, is located in Administrative Zone three of the Afar Region, above a gorge on the Awash River, after which the town is named. Awash Fentale District is a pastoral area in Afar National Regional State found 225 km northeast of Addis Ababa. The elevation of the area is between 736 and 801 m above sea level, of which 90% are pastoralists and 10% are agro-pastoralist areas. People in the region, therefore, depend mainly on livestock production for their livelihood [19]. The lowlands are generally characterized by relatively high temperatures, drought, and fragile arid and semiarid ecology [20].

### Collection and storage of soil samples

Isolation of nematophagous fungi was conducted from November 2019 to January 2020. To isolate the nematophagous fungi, 27 soil samples were randomly taken from each of the three different agro-ecological climates (9 from each agro-ecological climatic zone). For each study site, samples were collected from the soil of decomposed animal feces/dung, agricultural/farmlands, and forest lands in triplicates. The Geographic coordinates (location) and altitudes of the sampling site were measured by a Graphical positioning System (GPS) (see Additional file 1). From each site, approximately 250 g of soil sample was collected from the surface to the depth of 5 cm by an auger. To avoid cross-contamination, the auger was sterilized by dipping it in ethanol between sampling points. These sampled soils were collected in a closed sterile polythene bag and labeled, properly stored in the icebox, and brought to the laboratory, then processed within the subsequent day of collection [21].

### Isolation of nematophagous fungi

All laboratory works were conducted in the Microbiology laboratory located in the College of Veterinary Medicine and Agriculture in Addis Ababa University.

#### Culturing

Isolation of the nematophagous fungi was done using the soil sprinkle technique [22]. Using chloramphenicol-2% water agar (CHF-WA) medium, primary isolation of nematophagous fungi was achieved. The prepared agar was dispensed into 10 cm diameter Petri dishes. The 2 g soil samples were distributed in the center of Petri dishes containing the water–agar medium with 0.05% concentration of chloramphenicol. Then, 1 ml of live *Haemonchus contortus* third-stage larvae (obtained from Veterinary parasitological laboratory in College of Veterinary Medicine in Addis Ababa University) was added as bait, and the Petri dishes were incubated at 27 °C and monitored daily under a stereomicroscope for 10 days.

#### Sub-culturing

After 10 days of culturing, fragments of the culture were transferred to Petri dishes containing Potato Dextrose Agar (PDA) medium with chloramphenicol until pure fungal cultures were obtained [23]. Then a fragment of agar was cut from the periphery of actively growing culture using a sterile scalpel blade with a handle. Then the fragment was transferred to new plates, 1 ml of resuspended *Haemonchus contortus* larvae was added. Finally, we follow until the fungus forms a trapping structure and conidia. The nematophagous fungus was grown for two weeks in commercial PDA at 25 °C to detect conidia and morphology of trapping structure of nematophagous fungi species [10]. The morphology of the trapping device and conidia which were characterized by BRIC (2014) was used to aid the identification of nematophagous fungi. Actively growing cultures in Petridis plates were then examined under both the binocular dissecting microscope and the compound microscope. Characteristics such as nodular development, branching, and conidia were noted with this type of examination. Conidium shape was then determined by making temporary mounts in lactophenol-cotton blue.

### Staining

Lactophenol cotton blue is a stain that is used to examine fungal elements following either a tape preparation or a scraping. This stain contains phenol that kills the organisms, lactic acid that preserves fungal structures, and cotton blue that stains the chitin found in the fungal cell walls. The microscopic fungal morphology was used to identify nematophagous fungi. Four-step activities were conducted to accomplish such tasks. First, a drop of lactophenol cotton blue solution was added on a clean slide after that a small amount of fungal culture (mycelial mat) was removed from the edge (young colonies) by using one sterilized needle, and fungal culture was prepared on the slide by using the second needle to tease out the fungal structures then the coverslip was gently placed on the slide by lowering it down and avoiding air bubbles, finally the identification of nematophagous fungi species was conducted based on the morphology of trapping structures and conidia by high objective power of compound microscope [24, 25]. Material requirements and procedures for lactophenol cotton blue staining materials are available (see Additional file 2). In the end, the isolated fungi were identified at both the genus and species level based on their morphological characters and microscopic analysis.

### Determination of soil moisture

To determine the moisture content of the soil sample, two aluminum foils were prepared and their empty weight was taken. An aliquot of approximately 50 g of moist soil was placed into each aluminum foil and was reweighed. The soil was dried overnight at 105 °C in the oven. After allowing the dishes to cool, the soil sample within the dish was weighed to know the weight of the dry soil. Then the moisture content was calculated by the following formula [26].

$$\% \,moisture\,content\, = \,\tfrac{(Weight\,of\,moist\,soil)\,\, - \,\,(weight\,of\,dry\,soil)}{{Weight\,of\,dry\,soil}}$$

### Data analysis

Data on the presence or absence of the different types and numbers of nematophagous fungi was entered into Microsoft Office Excel 2007 software, and STATA 14 was used for descriptive analysis such as frequencies and percentages. Chi-square test was used to determine the presence of an association between soil sample type, soil moisture, and agro-ecological climates, and *P* values ≤ 0.05 were considered as a significant association.

## Results

### Isolation of fungal species

In this study, a total of thirty-three nematophagous fungal isolates of four genera and six fungal species were obtained from three different soil samples (dung, forest, and agricultural soil samples) taken from three different agro-ecological zones (Debre-Berhan, Bishoftu, and Awash) (Table 1).

**Table 1:** Distribution of isolated fungal Species from each location with Soil samples

Location	Dung soil	Agri-soil	Forest soil	Total
Awash	5	3	3	11
Bishoftu	4	4	4	12
Debre-Berhan	4	3	3	10
Total	13	10	10	33

**Fig. 2:**
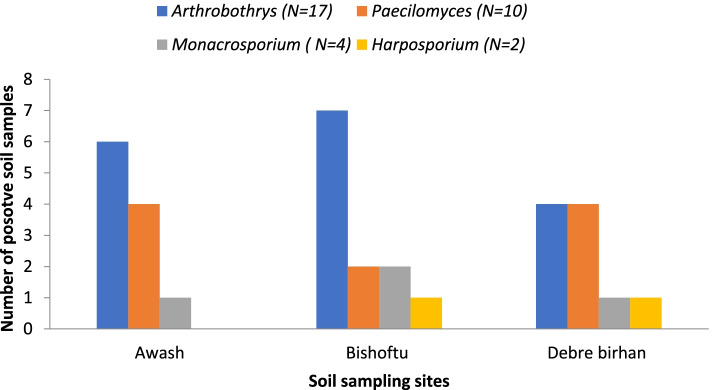
Occurrence of four genera of nematophagous fungi in three agro-ecologies

The four genera identified in this study were *Arthrobotrys, Paecilomyces, Monacrosporium, and Harposporium*. *Arthrobotrys* was the most widely isolated genera with an occurrence of 51.5% followed by *Paecilomyces* with an occurrence of 30.3%. Except for *Harposporium* which was not isolated from lowland samples (Awash), all the three genera prevail in all agro-ecologies. *Arthrobotrys* were more prevalent in soil samples from Awash and Bishoftu areas, which represent lowlands and mid-altitudes respectively (Fig. 2).

### Species of nematophagous fungi by agro-ecological zone and sample type

**Fig. 3:**
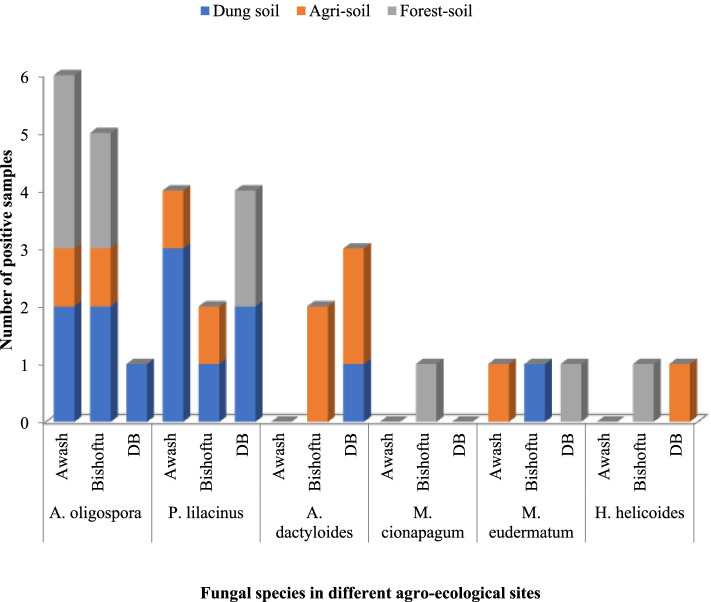
Distribution of fungal species by soil sample and study area

Based on the morphological characterization of fungal conidia, the six species of nematophagous fungus isolated in this study were *Arthrobotrys oligospora, A. dactyloides, Paecilomyces lilacinus, Monacrosporium cionopagum, M. eudermatum*, and *Harposporium helicoides*. *Arthrobotrys oligospora* was the most commonly detected species with an occurrence of 36.4% followed by *Paecilomyces lilacinus* with 30.3%. The dominant species from the lowland Awash and midland Bishoftu areas were *A. oligospora* and *P. lilacinus* whereas, in Debre-Berhan (highland), *P. lilacinus* and *A. dactyloides* were more available than others*. A. oligospora*was is commonly found in dung and forest soils in Awash and Bishoftu whereas *P. lilacinus* was better isolated from dung soil of Awash and dung and forest soils of the Debre-Berhan area. *A. dactyloides* is abundant in agri-soil of the highland category (Fig. 3).

### Association between soil moisture and isolated fungal species

**Fig. 4:**
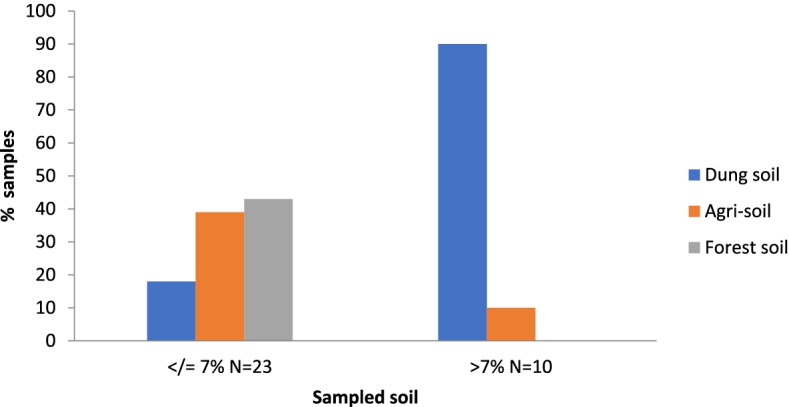
Distribution of fungal species by soil sample and soil moisture content

The moisture content of the soil samples ranged between 1% and 14.8% (2.2%-14.8% for dung soil, 1%-7.5% for agri-soil, and 1%-5.9% for forest soil), dung soil being at a higher frequency in the moisture content category of ≥ 7% (*P* = 0.0001, Fig. 4).

**Fig. 5:**
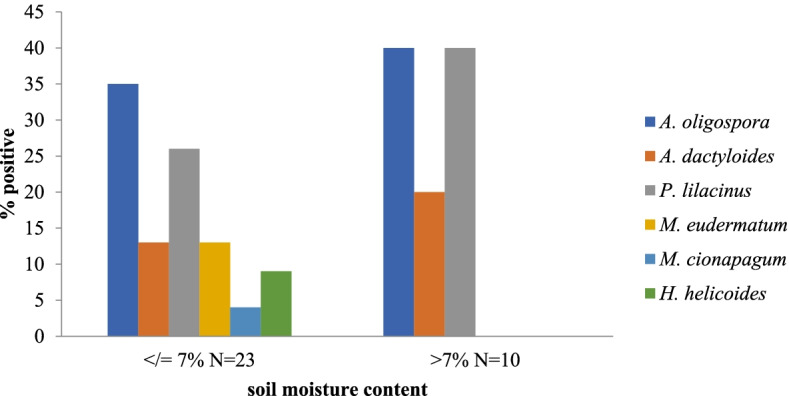
Distribution of fungal species by soil sample and soil moisture content

On the other hand, 23 (69.7%) fungal species were identified from soil samples that had moisture content less than or equal to 7% whereas the remaining 30.3% inhabited soils with moisture content above 7% (Fig. 5).

**Fig. 6:**
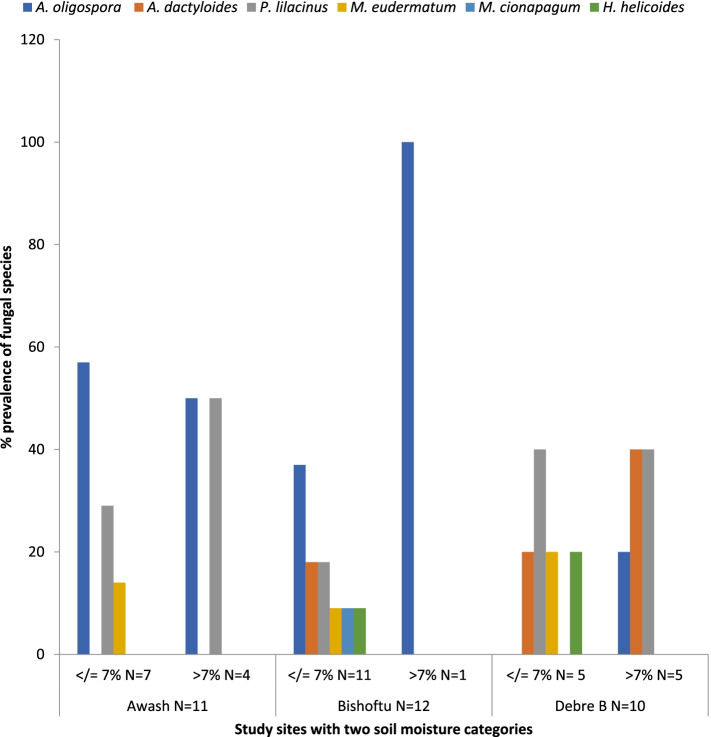
Prevalence of fungal species in study sites with two soil moisture categories

*A. oligospora* and *P. lilacinus* were common in both categories of soil moisture in general and in the lowland in particular (Fig. 6). Despite these facts, there is no appreciable effect of soil moisture on the occurrence of the different species of fungi (*P* > 0.05).

## Discussion

Soil harbors a diverse range of fungi and many of them are rivals of nematodes. At the same time, many economically important nematode parasites of livestock spend much of their life cycle in soil, foliage, or dung. In these environments, they are particularly vulnerable to a wide range of soil-borne nematophagous fungi that kill nematodes after they have trapped them, or have been ingested as spores [27].

Different study findings support our study (Table 2). This study has demonstrated that nematophagous fungi were widespread in occurrence and their diversity differs from one agro-ecological climate zone to the other. This is consistent with a study by *Gray NF*, which revealed nematophagous fungi have an extensive worldwide distribution, in all climates, and habitats [17]. It is also in line with a previous study from China [28–30] that reported the presence of nematophagous fungi in a wide range of environments.

**Table 2:** Previous study findings on the isolation of Nematophagus fungi

Author	Title	Findings
Niu XM and Zhang KQ	A model organism for understanding the interaction between fungi and nematodes	*Arthrobotrys oligospora* is the most common nematode-trapping fungus with the characteristic ability to form adhesive trapping nets once in contact with nematodes
Gray NF	Nematophagous fungi with particular reference to their ecology	*Hyphomycetes* is formed a complex three-dimensional adhesive network. With ***35*** species known, it is frequently encountered in nearly all types of soil The effect of the major soil variables such as soil moisture, organic matter, pH, nematode density, soil nutrients, and metals on the distribution of nematophagous fungi are proved
Saumell CA, et al	Nematophagous fungi from decomposing cattle feces in Argentina	Seventeen species from nine genera of nematophagous fungi are identified Twelve and three species are nematode-trapping fungi and endoparasitic fungi respectively *Arthrobotrys conoides*, *Arthrobotrys oligospora*, *Duddingtonia flagrans*, *Monacrosporium doedycoides, Arthrobotrys robusta*, and *Drechmeria coniospora* are the most frequently isolated species
Hao, Y et al	Ecology of aquatic nematode-trapping hyphomycetes in southwestern China	No species are isolated from the 20 samples collected from the bottom of Dianchi Lake, but from 980 samples 35 species are isolated, 21 of which are of the genus *Monacrosporium,* 8 are *Arthrobotrys,* and 6 are *Dactylella* The most common isolated species are *Arthrobotrys musiformis, A. oligospora, Monacrosporium ellipsosporum*, and *M. thaumasium*, which are isolated from 17, 18, 13, and 15 sites, respectively. *A. conoides* is the dominant species in Baoshan; *A. musiformis* is the dominant species in GeJiu and JingHong; *A. oligospora* was the dominant species in BaoShan, LanPing, Dianchi Lake and Panlong River; *M. ellipsosporum* was the dominant species in Wen- Shan and SiMao; and *M. megalosporum* is the dominant species in Lotus Pool. Of the 35 species, 17 produced adhesive networks, which amounted to 48.6% of the total isolated aquatic nematode-trapping hyphomycetes
Swe, A. et al	Nematode-trapping fungi from Arthrobotrys mangrove habitats	Seventeen nematode-trapping fungal species are identified from 480 composite samples at eight different mangrove sites The most common species in mangroves were *Monacrosporium thaumasium*, *A. oligospora* and *Monacrosporium eudermatum*, Twenty-four nematode-trapping fungal species are isolated from 540 composite samples collected at nine different terrestrial sites. The most common species are *A. oligospora*, *Arthrobotrys musiformis*, and *M. thaumasium* Twenty nematode-trapping fungal species are isolated from 300 composite samples collected from five different rivers and streams. The most common species were *M. eudermatum*, *A. oligospora*, *M. thaumasium*, and *Arthrobotrys musiformis* Thirty-one nematode-trapping are isolated, which consists of 13 *Arthrobotrys*, 15 *Monacrosporium*, and 3 *Dactylella* species. Twenty-six species were rare Twenty-nine of the species reported in the present study are new to Hong Kong. Seventeen species isolated from mangrove are new from marine habitats
Shams Ghahfarokhi M. et al	Isolation and Characterization of the Nematode-Trapping Fungus Arthrobotrys oligospora	11 nematophagous fungi are isolated from 150 pasture soil samples based on the observation of characteristic conidia and traps around the immobilized larvae. From these, 3 pure cultures were made and identified as *A. oligospora* A. oligospora and D. flagrans kill the third-stage larvae of H. contortus at a concentration of 20 × 10^3^ onidia/g feces
Durand, D.T et al	Survey of nematophagous fungi in South Africa	Five isolates of *D. flagrans* and 73 isolates of other nematophagous fungi were obtained from 384 cultures of soil, feces, compost, leaf litter, and aqueous suspensions of infective larvae contaminated with unidentified fungi The most common nematophagous fungus isolated is *Arthrobotrys oligospora* The other nematophagous fungi isolated were *Arthrobotrys superba*, *Arthrobotrys dactyloides*, *Arthrobotrys botryospora*, *Arthrobotrys scaphoides* and *Monacrosporium gephyropagum* Two isolates of *D. flagrans* are isolated from compost and three from leaf litter
Farrell, F.C. et al	The nematode-trapping fungus Arthrobotrys oligospora in soil of the Bodega marine reserve: distribution and dependence on nematode parasitized moth larvae	*A. oligospora* is detected in 39 of 42 lupine samples and 32 of 42 nonlupine samples Nematode-trapping fungi detected in the laboratory experiment included those that form adhesive networks (A. eudermata, A. musiformis, A. paucispora), adhesive branches (G. gephyropagum), constricting rings (D. doedycoides), and adhesive knobs (N. concurrens); population densities of these fungi did not exceed 39 propagules per g of soil in any arena or 5 propagules per g of soil for any treatment
Wachira, P.M. et al	Influence of land use and soil management practices on the occurrence of nematode destroying fungi in Taita Taveta, Kenya	Organic inputs (cow manure and chicken manure) significantly affected the occurrence of nematode destroying fungi in the study area. Inorganic inputs (chemical fertilizers and pesticides) did not show any effect on the occurrence of nematode destroying fungi Land use significantly affected the occurrence of nematode destroying fungi. The land use explained 63.73% of the observed absence or presence of nematode All the isolates of nematode-trapping fungi were twenty-eight in number and after identification they were grouped into three genera, *Arthrobotrys, Monacrosporium*, and *Nematoctonus*. The genus *Arthrobotrys* is isolated in all the land uses except in fallow. The genus Monacrosporium is isolated in four land uses; vegetables maize, Napier, and fallow while Nematoctonus occurred only in maize bean. *A. oligospora, A.dactyloides,* *M.cionapoagum, Monacrosporium sp Nematoctononus sp occurred in frequencies of 42.9, 28.6, 17.9, 7.1, and 3.6% respectively* A.oligospora formed adhesive nets, non-constricting rings, and three-dimensional structures which caught nematodes and consumed them within twelve hours The number of traps increased with an increased number of nematodes reaching the highest pick on the eighth day, which also increased the number of trapped nematodes
Wairimu WJ. et al	Diversity of naturally occurring nematode destroying fungi and their interaction with soil amendments in banana farms in Meru and Embu counties	Fifty-eight isolates of nematode destroying fungi, distributed in five genera and six taxa were identified in this study. The species *Arthrobotrys oligospora* (Fresen), *A. dactyloides* (Drechsler), *Monacropsporium cionopagum* (Subramanian), *Meria coniospora* (Drechsler), *Dactyllela lobata (Duddington)*, and *Harposporium aungullilae* (Lohde) are identified. All the nematode destroying fungi are significantly affected by the ecological zone. The lower zone had a mean occurrence of nematode destroying fungi of 2.8, with 1.8 and 1.2 being recorded in middle and high zones, respectively. The lower zone represented 48.3% of the total nematode destroying fungi. Out of all the isolates obtained, 48.3% are from the lower zone *Monacrosporium cionopagum* was the most frequently isolated species of nematode destroying fungi while *Harposporium aungulliale* is the last one
Yang, Y et al	Evolution of nematode-trapping cells of predatory fungi of the Orbiliaceae based on evidence from rRNA-encoding DNA and multiprotein sequences	**Phylogenetic Relationship of Trapping Devices** Cladograms based on parsimony analyses of nucleotide sequences of rDNA ITS regions and the combined data set of four genes (ITS, *bt*, *rpb*, and *ef1-α*) revealed similar topological structures The ML tree based on the combined data set of 2,706 bp provided more detailed information [high bootstrap values as assessed by 1,000 minimal evolution (ME) bootstrap replications] than the trees based on rDNA in the ITS region and revealed distinctive signatures that were diagnostic for different trapping devices. The data resulted in two main clades representing two different trapping mechanisms (adhesive and nonadhesive). The nonadhesive clade [98% bootstrap support value (BSV)] consists of species with CR and was paraphyletically evolved with the adhesive clade, including trapping of a knob, stalked knob, hyphal column, NCR, and network. The evolution of the adhesive trapping structures with the same trapping mechanism was resolved with the combined data-set tree. Two subclades corresponding to the AN (100% BSV). and other adhesive structures (63% BSV) were strongly supported. AC, AK, and AK associated with NCR grouped in the same subclade, suggesting their close phylogenetic relationship **Phylogenetic Relationship of Adhesive Trapping Devices** In the subclade of AK and column-trapping devices, eight strains forming AC clustered into one group with a 98% BSV and diverged from the other adhesive trapping devices. The species forming sessile or short-stalked knobs (*Dactylellina parvicollis*, *Dactylellina phymatopaga*, *Dactylellina querci*, *Dactylellina haptospora*, and *Dactylellina tibetensis*), representing the primitive character states, were separated early from other species. The species forming adhesive short-stalked (*Dactylellina drechsleri*, *Dactylellina entomopaga*, *Dactylellina mammillata*, and *Dactylellina ellipsospora*) comprised a subgroup with a 78% BSV. Species with long stalked knobs (*Dactylellina copepodii*, *Dactylellina haptotyla*, and *Dactylellina leptospora*) were associated with NCR and are clustered into the other subgroup with a 70% BSV **Ancestral State Reconstruction** Six characters (five trapping device types and no traps), each with two states (present, absent), were calculated by tracing all changes, and a tree with a tree length of 8 was generated. The evolution of the CR went through two stages. One was the formation of the stalks, and the other was the formation of the rings. During the evolution of the adhesive traps, each trap got one change from its ancestor. The primogenitor of the trapping device first obtained an adhesive strategy and formed AN. Afterward, the evolution focused on covering one specialized cell (sessile knob or protuberance) with adhesive materials. The protuberance proliferated to form the AC. The sessile knob developed an extended stalk to form stalked knob, and some species reproduced several adhesive cells, which might be the origination of NCR
Jafee BA et al	Wood, nematodes, and the nematode-trapping fungus Arthrobotryes oligospora	Wood mass loss (decomposition) on agar is enhanced when both large numbers of nematodes *S. glaseri* and fungus *A. oligospora* are added Wood mass loss in the soil is affected by the addition of KNO3
Elshafie, A.E. et al	Diversity and trapping efficiency of nematophagous fungi from Oman	A survey of the nematophagous mycobiota biodiversity of 82 soil and leaf-litter samples in the Sultanate of Oman yielded ten species of nematode-trapping fungi belonging to three genera. The species are: *Arthrobotrys eudermata*, *A. thaumasia*, *A. musiformis*, *A. oligospora, A. oligospora var. oligospora, A. oudemansii, A. multiformis,* *A. javanica, Drechslerella brochopaga* and *Gamsylella geophyropaga*
Mo M.H et al	Diversity and metal tolerance of nematode-trapping fungi in Pb-polluted soils	The diversity of nematode-trapping fungi (NTF) in two lead (Pb) mines in Yunnan Province, China was investigated in 2004. In total, 20 species belonging to five genera are identified from 500 samples collected at the Lanping and the Huize mines. Pb concentrations ranged from 216–7,150 mg/kg for the former and 132–13,380 mg/kg for the latter, respectively. The fungi are divided into five groups based on different trapping mechanisms. The trapping-net producer group contained the largest number of species, with nine. Two predators, *Dactylellina ellipsosporum* and *Arthrobotrys oligospora*, were found at frequencies of 32.85% and 15.41%, respectively. The diversity indexes of NTF were positively correlated with Pb pollution levels in both the Lanping Mine (r = 0.66) and the Huize Mine (r = 0.72. For most strains of a given species, there was no significant difference (*P* > 0.01) in the Pb tolerance between the strains isolated from habitats with low or high Pb concentrations
Xiang, M et al	Effect of environment on the aboundance and the activity of the nematophagous fungi Hirsutella Minnesotensis in soil	**Effect of soil moisture** The quantity of *H. minnesotensis* DNA in the soil as determined by real-time PCR was highest at 5 and 10 °C, sharply declined between 10 and 15 °C, gradually declined between 15 and 20 °C, and did not change from 20 to 30 °C. *Hirsutella minnesotensis* is not detected by real-time PCR in soil tubes that were not inoculated with the fungus **Influence of soil texture** There were no significant differences in the percentage of parasitized J2 in the native soil, in soil amended with 10–70% fine soil particles, and in soil amended with 10% sand (*P* = 0.083), whereas the percentage of parasitized J2 decreased with the increase of sand in the soils (30%, 50%, 70% sand added) (*P* < 0.001). In contrast, the quantity of *H. minnesotensis* DNA was highest in the native soil, and in soil amended with 30%, 50%, and 70% fine soil particles (*P* = 0.359). Increasing the ratio of silicon dioxide sand to native soil resulted in a dramatic decline in the quantity of *H. minnesotensis* DNA and *H. minnesotensis* parasitic activity. However, the addition of 10% fine soil particles to the soil decreased the quantity of DNA but not the activity of the fungus relative to the native soil. Decreasing the ratio of fine soil particles to native soil from 70 to 10% reduced the quantity of DNA but not the parasitic activity

In this study, four major genera, *Arthrobotrys*, *Paecilomyces, Monacrosporium,* and *Harposporium* were isolated from the three agro-ecologies and three soil sources. Studies from different countries support our study findings [23, 31].

The study at hand revealed that *Arthrobotrys oligospora*was are the most dominant species of nematophagous fungus. It had the characteristics of the ability to form adhesive trapping nets when in contact with nematodes. It was isolated from compost/decomposed dung soil, agricultural soil, and forest soil with an overall frequency of 36.36%. This result may be indicating that *Arthrobotryes oligospora* were best adapted to the biotic and abiotic conditions of many areas. Another possible reason may be related to its high saprophytic ability and the increased agricultural intensification caused by soil disturbance and the addition of fertilizers. Similar results have been reported in South Africa [32, 33], Kenya [34, 35], China [14, 36–38], and Oman [39].

The greatest diversity of nematophagous fungi species was recorded in midland (Bishoftu). This is maybe due to suitable/optimum environmental conditions (temperature, rainfall, moisture, and light) and land morphology. From low land (Awash), they are least diverse perhaps due to their high temperature, drought, scarce rainfall, fragile arid and semi-arid ecology, and minimum land disturbance/ cultivation or far miming activity. In Debre Berhan, the nematophagous species were more diverse than lowland/Awash and less diverse than the midland due to the conducive environmental condition [35].

This study also examined the relation of soil type and soil moisture on the distribution of nematophagous fungi. The interaction of soil type with nematophagous fungi species indicated that the highest percentage was obtained from dung soil, which is known to be rich in organic matter. This is maybe due to the presence of macronutrients (Nitrogen, Phosphorus, and Potassium) from compost and the presence of nematodes that are excreted with animal feces.

In this study, *Arthrobotryes oligospora* was the most abundant in forest and dung soils, compared to agricultural soil suggesting an abundance of organic matter in such soils and the preference of these conidia forming fungal species for such types of soils. As different scholars revealed [38, 40], Nitrogen, Phosphorous, Potassium, Iron and nematode density affect the distribution of nematophagous fungal species.

The current study has demonstrated that the moisture content of the soil sample had no significant difference with the fungal species isolated. The dominant species, *Arthrobothrys oligospora* was the most common in both categories of soil moisture examined. Such characteristics vary with species of the fungi [41], working on fungal species *Hirsutella Minnesotans* have reported that the species has a greater potential to multiply and control pest nematodes in cooler, drier, and heavier soils.

Despite it being the first, this study is not without limitations since we did not assess the molecular characterization and the efficacy of the nematophagous fungi. Therefore, further experimental studies should be conducted on the identification, molecular characterization, and efficacy of nematophagous fungi. Besides, optimum growth conditions should be studied for mass culturing of nematophagous fungi to use for the commercial purpose of biological controlling of nematode parasites. Furthermore, when evaluating fungal prevalence at soil moisture level, we did not take into account other environmental parameters such as soil organic matter, carbon, and other nutrients.

## Conclusion

The study has confirmed that nematophagous fungi were widely distributed in the study agro-ecological climatic zones but differ in their diversity. The in vitro experimental study was conducted from PDA and WA (2%) by using *Haemonchus contortus* as the bait. This study isolates a total of 33 nematophagous species which are grouped into four genera and six species. The genera were *Arthrobotrys, Paecilomyces, Monacrosporium,* and *Harposporium*. On the other hand, the species identified were *Arthrobothryes oligospora, Arthrobotrys dactyloides, Harposporium helicoides*, *Monacrosporium cionopagum, Monacosporum eudermatum,* and *Paecilomyces lilacinus*. Among these, the distribution of *Arthrobothryes oligospora* was the highest but *Monacosporum cionopagum* was the lowest. The interaction of soil type with nematophagous fungi species indicated the highest percentage was obtained from dung soil. This study has demonstrated that the *Arthrobotrys oligospora* was the most common in each category of soil moisture examined. However, the moisture content of the soil sample had no significant difference with the fungal species isolated. Further experimental studies on the identification, molecular characterization, and efficacy of nematophagous fungus are recommended by the authors. Furthermore, optimal growth conditions for mass culturing of nematophagous fungi should be investigated to employ them commercially for biological nematode parasite control.

## Supplementary Information

**Additional file 1: Supplementary file 1.** Geographic coordinates (GPS location) andaltitude of the sampling site.

**Additional file 2: Supplementary File 2.** Material requirement and procedures for lactophenol cotton bluestaining materials.

**Additional file 3: Supplementary file 3. **Microscopic morphology of isolated nematophagous fungal conidia(A) Arthrobotryes oligospora (pear-shapedtwo celled conidia, distal cell is smaller than the proximal); (B) Monacosporium eudermatum (ellipsoidal conidia with sharp ended);(C) Monacrosporium cionopagum(apicalmulticellular conidia with somewhat arrow ends); (D) Harposporium helicoides(curved conidia with barbed ends); (E) Paecilomyces lilacinus (Conidia areellipsoid in shape and are single celled conidiophores develop in group oflateral branches from which each 2-4 bottle shaped phialides grow); (F) Arthrobotrys dactyloides (two celledconidia, proximal and distal cells has almost equal size), (G) L3 of Haemonchus contortus trapped by nematode trapping fungi.

## Data Availability

All result-based data is in the paper and anyone can access the data set from the corresponding author (maradonaber02@gmail.com).

## Abbreviations

AR: Anthelmintic Resistance
AOL: *Arthrobotrys* *oligospora* Lectin
BCA: Biological Control Agent
BW: Body Weight
CSA: Central Statistical Agency
CMA: Corn Meal Agar
CHF-WA: Chloramphenicol Water Agar
ECM: Extracellular Matrix
GIN: Gastro-Intestinal Nematodes
GIT: Gastro-Intestinal Tract
NMSA: National Metrology Service Agency
NTF: Nematode Trapping Fungus
PCR: Polymerase Chain Reaction
PDA: Potato Dextrose Agar
WA: Water Agar
