# Supplementary material for: Isolation of nematophagous fungi from soil samples collected from three different agro-ecologies of Ethiopia
Source: BMC Microbiol. 2022 Jun 17;22:159. doi: 10.1186/s12866-022-02572-4 (PMC9204992; doi:10.1186/s12866-022-02572-4)
Supplement: Supplementary file 1 — Additional file 1: Supplementary file 1. Geographic coordinates (GPS location) andaltitude of the sampling site. [file 12866_2022_2572_MOESM1_ESM.docx]

**Supplementary file 1**: Geographic coordinates (GPS location) and altitude of the sampling site

| (Study areas) | Dung soil | | Forest soil | | Agri-soil | |
| --- | --- | --- | --- | --- | --- | --- |
|  | Location | Elevation | Location | Elevation | Location | Elevation |
| Awash | N 08^0^59.967'  E040^0^10.623' | 914m | N 08^0^52.952'  E038^0^46.657' | 868m | N 09^0^19.288'  E040^0^10.022' | 741m |
| Awash | N 08^0^59.538'  E040^0^10.277' | 530m | N 08^0^59.982'  E040^0^10.729' | 899m | N 09^0^19.214'  E040^0^11.055' | 337m |
| Awash | N 08^0^59.448'  E040^0^10.123' | 922m | N 08^0^59.997'  E040^0^10.730' | 896m | N 09^0^19.248'  E040^0^11.198' | 336m |
| Bishoftu | N 08^0^45.783'  E038^0^49.949' | 1814m | N 08^0^46.012'  E038^0^59.850' | 1890 | N 08^0^46.316'  E039^0^00.750' | 1881 |
| Bishoftu | N 08^0^46.346'  E038^0^59.964' | 1894m | N 08^0^45.994'  E038^0^59.798' | 1891 | N 08^0^46.195'  E039^0^00.520' | 1880 |
| Bishoftu | N 08^0^ 46.562'  E039^0^ 00.174' | 1895m | N 08^0^45.986'  E038^0^59.794' | 1887 | N 08^0^46.186'  E039^0^00.516' | 1879 |
| Debre-Berhan | N 09^0^42.275'  E039^0^33.590' | 2812m | N 09^0^41.942'  E039^0^33.631' | 2800 | N 09^0^41.338'  E039^0^33.137' | 2764 |
| Debre-Berhan | N 09^0^42.243'  E039^0^33.511' | 2815m | N 09^0^42.208'  E039^0^33.231' | 2806 | N 09^0^41.729'  E039^0^33.660' | 2790 |
| Debre-Berhan | N 09^0^42.371'  E039^0^33.531' | 2828m | N 09^0^42.147'  E039^0^33. 186' | 2795 | N 09^0^42.067'  E039^0^33.600' | 2801 |
