# Supplementary material for: Isolation of nematophagous fungi from soil samples collected from three different agro-ecologies of Ethiopia
Source: BMC Microbiol. 2022 Jun 17;22:159. doi: 10.1186/s12866-022-02572-4 (PMC9204992; doi:10.1186/s12866-022-02572-4)
Supplement: Supplementary file 2 — Additional file 2: Supplementary File 2. Material requirement and procedures for lactophenol cotton bluestaining materials. [file 12866_2022_2572_MOESM2_ESM.docx]

**Supplementary File 2**. Material requirement and procedures for lactophenol cotton blue staining materials

**Materials**

- Young fungal culture
- Glass slide Cover slip
- Needle/wire loop and straight wire
- Lactophenol cotton blue
- Bunsen burner

**Procedures:**

1. Take aclean grease free slide

2. Add adrop of mounting tube that is phenol cotton blue solution on aslide

3. Sterilize the needle cool it then transfer mycelian mat on fluid and press it gently so that it easily mix with the stain

4. The clean cover slip is gently placed on the slide by lowering it down and avoiding air bubbles

5. Take ablotting paper and wipe the excess stain

6. Observe under the lower x10 objective to locate to the object and using the high power x40 objective of microscope to confirm the presence of fungal structures (micro/macro conidia, spore and hyphae)
