# Supplementary material for: Isolation of nematophagous fungi from soil samples collected from three different agro-ecologies of Ethiopia
Source: BMC Microbiol. 2022 Jun 17;22:159. doi: 10.1186/s12866-022-02572-4 (PMC9204992; doi:10.1186/s12866-022-02572-4)
Supplement: Supplementary file 3 — Additional file 3: Supplementary file 3. Microscopic morphology of isolated nematophagous fungal conidia(A) Arthrobotryes oligospora (pear-shapedtwo celled conidia, distal cell is smaller than the proximal); (B) Monacosporium eudermatum (ellipsoidal conidia with sharp ended);(C) Monacrosporium cionopagum(apicalmulticellular conidia with somewhat arrow ends); (D) Harposporium helicoides(curved conidia with barbed ends); (E) Paecilomyces lilacinus (Conidia areellipsoid in shape and are single celled conidiophores develop in group oflateral branches from which each 2-4 bottle shaped phialides grow); (F) Arthrobotrys dactyloides (two celledconidia, proximal and distal cells has almost equal size), (G) L3 of Haemonchus contortus trapped by nematode trapping fungi. [file 12866_2022_2572_MOESM3_ESM.docx]

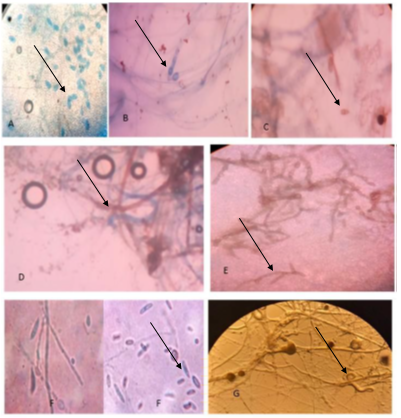


**Supplementary file 3**. Microscopic morphology of isolated nematophagous fungal conidia

(A) Arthrobotryes oligospora (pear-shaped two celled conidia, distal cell is smaller than the proximal); (B) Monacosporium eudermatum (ellipsoidal conidia with sharp ended); (C) Monacrosporium cionopagum(apical multicellular conidia with somewhat arrow ends); (D) Harposporium helicoides(curved conidia with barbed ends); (E) Paecilomyces lilacinus (Conidia are ellipsoid in shape and are single celled conidiophores develop in group of lateral branches from which each 2-4 bottle shaped phialides grow); (F) Arthrobotrys dactyloides (two celled conidia, proximal and distal cells has almost equal size), (G) L3 of Haemonchus contortus trapped by nematode trapping fungi.
